# Mechanism of Gujian Tiaosui Decoction in the treatment of osteoarthritis: Based on network pharmacological analysis and Mendelian randomization

**DOI:** 10.1097/MD.0000000000045071

**Published:** 2025-10-10

**Authors:** Yiwen Zhu, Xiaoming Wang, Xiao Xiao, Jiao Situ, Qinguang Xu, Jieji Zhang, Wenjie Xu, Denghui You, Yong Ju, Yi Zhou, Jining Jiang, Peijian Tong, Shirong Yang

**Affiliations:** a Institute of Orthopaedics and Traumatology, The First Affiliated Hospital of Zhejiang Chinese Medical University, Hangzhou, Zhejiang, China; b Fenghua Hospital of Traditional Chinese Medicine, Ningbo City, Zhengjiang, China; c Guangyuan Hospital of Traditional Chinese Medicine, Guangyuan City, Sichuan, China; d Huzhou Central Hospital, Fifth School of Clinical Medicine Zhejiang Chinese Medical University Central Hospital, Huzhou City, Zhengjiang, China; e Xiwu Branch of Fenghua District Hospital of Traditional Chinese Medicine Medical Community, Ningbo City, Zhengjiang, China.

**Keywords:** GTD, Mendelian randomization, molecular docking, network, OA

## Abstract

Osteoarthritis (OA) is widely recognized as a common degenerative joint disease that imposes a significant burden on patients and society. Gujian Tiaosui Decoction (GTD), an empirical formula from Zhejiang Provincial Hospital of Traditional Chinese Medicine, has demonstrated clinical efficacy in treating OA. However, the therapeutic mechanisms underlying GTD’s effects on OA remain unclear. Potential targets of GTD and OA-related targets were identified using the TCMSP, OMIM, and GeneCards databases. A visual network of “GTD–compounds–key targets–pathways–OA” was constructed. Gene ontology and Kyoto encyclopedia of genes and genomes analyses were performed to explore biological processes and pathways. Key targets were further screened using Mendelian randomization and expression analyses. Molecular docking was conducted between key bioactive compounds and targets to identify signaling pathways and proteins associated with GTD’s therapeutic effects on OA. A total of 160 compounds with 575 unique targets related to GTD were retrieved from Traditional Chinese Medicine Systems Pharmacology Database and Analysis Platform, and 185 overlapping targets between GTD and OA were identified. Protein–protein interaction network analysis revealed core targets including AKT serine/threonine kinase 1, hypoxia-inducible factor-1α (HIF1A), estrogen receptor 1, SRC proto-oncogene, non-receptor tyrosine kinase, epidermal growth factor receptor, mitogen-activated protein kinase 3, matrix metalloproteinase 9, and signal transducer and activator of transcription 3. Gene ontology and Kyoto encyclopedia of genes and genomes enrichment analyses indicated that relevant biological processes involved in GTD’s anti-OA effects may include inflammatory response, positive regulation of protein kinase B signaling, protein phosphorylation, integral component of membrane, and protein serine/threonine/tyrosine kinase activity. Mendelian randomization analysis suggested a positive causal relationship between OA and 2 genes: HIF1A and IMPDH2. Molecular docking of 5 active ingredients with HIF1A and inosine-5′-monophosphate dehydrogenase 2 showed strong binding affinity. This study reveals the multicomponent, multi-target, and multi-pathway mechanism of GTD in the treatment of OA, providing a foundation for further experimental validation and suggesting new research directions.

## 1. Introduction

Osteoarthritis (OA) is a degenerative joint disease characterized by progressive cartilage degradation, aberrant subchondral bone remodeling, and chronic pain.^[1,2]^ As one of the most prevalent disabling conditions among the global elderly population,^[3]^ OA continues to impose an escalating disease burden. Accelerated global population aging has contributed to a sustained rise in OA incidence, with modeling projections indicating that OA will become the fourth leading cause of disability worldwide by 2050.^[4]^ Currently, there are no disease-modifying therapies available for OA in clinical practice. Although nonsteroidal anti-inflammatory drugs provide symptomatic pain relief, their long-term use is associated with serious adverse effects, including gastrointestinal hemorrhage, increased cardiovascular risks, and renal impairment.^[5,6]^ These complications significantly impair patients’ quality of life and impose substantial socioeconomic burdens. Consequently, identifying safe and effective interventions to mitigate OA progression represents a critical unmet medical need.

In recent years, Traditional Chinese Medicine (TCM) has demonstrated distinctive potential in the conservative management of OA. Rooted in the foundational TCM theory that “the kidney governs bones and produces marrow,” scholars have proposed the “Marrow Disease Theory.” This paradigm posits that bone disorders originate from functional abnormalities of “marrow” (specifically, the dysregulation of bone marrow mesenchymal stem cells and their microenvironmental homeostasis).^[7,8]^ The central tenet, “all bone diseases originate from marrow disorders,” emphasizes 2 therapeutic strategies: “internal marrow regulation,” which involves activating endogenous stem cell migration to lesion sites for tissue repair and homeostasis restoration, and “external marrow replenishment,” which refers to exogenous supplementation interventions. Guided by this theoretical framework, Gujian Tiaosui Decoction (GTD), an empirical formula that has been clinically applied for years at Zhejiang Provincial Hospital of Traditional Chinese Medicine, has demonstrated promising efficacy in alleviating OA symptoms and retarding joint degeneration. Nevertheless, its precise molecular targets and underlying pharmacological mechanisms remain incompletely characterized, limiting its modern application and international recognition.

The primary challenge in elucidating the mechanisms of complex TCM formulas lies in their intricate chemical composition, numerous interacting targets, and the inadequacy of single-target models. Network pharmacology, an emerging interdisciplinary methodology that integrates systems biology, network science, and computational informatics, provides a powerful framework for systematically deciphering the mechanisms of herbal formulations.^[9]^ This approach adopts a holistic “multi-component, multi-target, multi-pathway” perspective, constructing compound–target–disease interaction networks to identify core target clusters and key signaling pathways modulated by TCM interventions. Molecular docking technology further complements these predictions by computationally simulating binding modes and affinity between bioactive compounds and potential target proteins at the structural biology level.^[10]^ However, conventional network pharmacology primarily identifies associations and inherently lacks the capacity to infer causal relationships between specific targets and disease pathogenesis, representing a fundamental methodological limitation.

To address this constraint, Mendelian randomization (MR) has emerged as an innovative approach for causal inference.^[11]^ MR utilizes genetic variants strongly associated with an exposure (e.g., specific gene expression levels) as instrumental variables (IVs). Under conditions approximating randomization, MR infers causal relationships between the exposure and disease outcomes, thereby mitigating confounding factors and reverse causation biases inherent in observational studies. Drug-target MR, a major extension of MR, evaluates the potential causal effects of candidate drug targets (assessed via gene expression or protein abundance proxies) on disease risk, providing robust genetic evidence for target discovery and validation.^[12]^ In OA research, the causal relationships between key targets and OA pathogenesis or progression remain largely unexplored. Therefore, integrating network pharmacology (for target and pathway discovery) with drug-target MR (for causal inference and validation) presents a valuable strategy for uncovering novel OA therapeutic targets and deciphering the mechanisms of TCM formulas.

This study aims to systematically elucidate the key molecular targets and regulatory mechanisms underlying GTD’s intervention in OA by integrating network pharmacology and drug–target MR analyses. The research will first identify GTD’s bioactive components and their potential targets using public databases, followed by constructing a compound–target–OA interaction network to preliminarily identify core regulatory genes. Subsequently, MR will be rigorously employed to assess the causal relationships between these core targets and OA risk. Finally, molecular docking will validate the binding capability and modes between GTD’s key bioactive constituents and the core target proteins. This integrated approach seeks to delineate the modern pharmacological basis of GTD for OA treatment, establish a scientific foundation for TCM interventions in degenerative bone and joint diseases guided by the “Marrow Disease Theory,” and facilitate the development of novel OA therapeutics targeting the identified pathways.

## 2. Materials and methods

### 2.1. Screening of GTD chemical compositions and corresponding targets

The TCMSP database (https://old.tcmsp-e.com/tcmsp.php) was searched using the following herbal ingredients of GTD as keywords: Astragalus, Salvia Miltiorrhiza, Eucommia, Angelica, Cuscuta, Corydalis Yanhusuo, Achyranthes, Cooked Rehmannia, and Poria Cocos. Potential active ingredients from these 9 herbs were screened based on absorption, distribution, metabolism, and excretion properties. Compounds meeting the criteria of oral bioavailability ≥ 30% and drug-likeness ≥ 0.18 were selected. Corresponding target proteins of these components were collected. Additionally, active ingredients were supplemented through a review of relevant literature to construct a comprehensive GTD active ingredient database. The two-dimensional structures of the compounds were downloaded in SDF format from PubChem (https://pubchem.ncbi.nlm.nih.gov/), converted into 3D conformations using Chem3D (2020 version), and energy-minimized via molecular mechanics optimization. Processed proteins and ligands were converted to.pdbqt format using AutoDock Tools 1.5.6 (The Scripps Research Institute, La Jolla).

### 2.2. Construction of OA target database

The GeneCards (https://www.genecards.org/) and OMIM (https://www.omim.org/) databases were queried using “osteoarthritis” as the keyword to retrieve disease-related targets. Duplicates were removed to construct the OA target database.

### 2.3. Screening of active ingredients and disease common targets

The UniProt database (https://www.uniprot.org/) was used to standardize target protein gene names with the species set to “Human.” The Venny 2.1 tool (https://bioinfogp.cnb.csic.es/tools/venny/) was then used to identify overlapping targets between GTD active ingredients and OA-related targets.

### 2.4. Construction of protein–protein interaction (PPI) networks

To elucidate the correlation between active ingredient-related proteins of GTD and osteoarthritis-related targets, as well as to identify potential therapeutic targets of GTD for osteoarthritis treatment, the overlapping targets were imported into the STRING database (https://string-db.org/).^[13]^ A PPI network model was constructed with the biological species set as “Homo sapiens” and the minimum interaction confidence score set to “0.900” (highest confidence). The remaining parameters were kept as default. The resulting PPI network was then imported into Cytoscape software (version 3.9.1, The Cytoscape Consortium, San Diego ) for visualization and further analysis. To identify core targets within the PPI network, network topology analysis was performed using the CytoHubba plugin in Cytoscape. Multiple centrality measures (including degree centrality, betweenness centrality and closeness centrality) were calculated to evaluate the importance of each node. Nodes were ranked based on their centrality values, and those consistently ranked in the top 20% across at least 3 centrality measures were defined as core targets. In the visual representation of the PPI network, node size and color intensity were proportional to the degree centrality value, while the thickness of edges corresponded to the combined confidence score from the STRING database.

### 2.5. Construction of GTD active ingredient–target–disease network map

Active ingredient targets and disease targets were input into Venny 2.1 (https://bioinfogp.cnb.csic.es/tools/venny/) to generate Venn diagrams. An active ingredient–target–disease network was constructed and analyzed using Cytoscape 3.9.1.

### 2.6. Gene ontology (GO) function and Kyoto encyclopedia of genes and genomes (KEGG) pathway enrichment analysis

Core targets of GTD for OA treatment were analyzed using the Metascape database (http://metascape.org/), with the species set to “Homo sapiens” and a significance threshold of *P* < .01. GO functional and KEGG pathway enrichment analyses were performed to identify key biological processes (BP) and pathways. A component–target–pathway network was visualized using Cytoscape 3.9.1.

### 2.7. Mendelian randomization

All data used in this study were obtained from the IEU OpenGWAS database (https://gwas.mrcieu.ac.uk/), a comprehensive repository that provides access to summary data from genome-wide association studies (GWAS) conducted worldwide. This database aggregates a large amount of GWAS data from multiple research consortia, encompassing genetic associations for various diseases, physical traits, and other complex phenotypes. Specifically, OA GWAS data were obtained from the study by Dönertaş et al^[14]^ (accession number: ebi-a-GCST90038686; https://opengwas.io/datasets/ebi-a-GCST90038686), which included 484,598 participants (39,515 cases and 445,083 controls), with OA diagnosis based on ICD-10 criteria. We selected expression quantitative trait loci (eQTL) data from the eQTLGen consortium (https://www.eqtlgen.org/) as the exposure, and the OA dataset (ebi-a-GCST90038686) as the outcome. A two-sample MR framework was applied to investigate the causal association between gene expression levels and OA risk, using single nucleotide polymorphisms as IVs. Genetic instruments were selected from publicly available eQTL data based on the following criteria: single nucleotide polymorphisms were significantly associated with the exposure (*P* < 5 × 10⁻^⁸^); linkage disequilibrium (LD) was clumped using a window size of 10,000 kb and an *r*² threshold of 0.001 to ensure independence among IVs; weak IVs were excluded using an *F*-statistic threshold > 10. MR analyses were performed using the “TwoSampleMR” package in R, with the inverse variance weighted (IVW) method serving as the primary statistical approach to assess the causal effect of gene expression on OA risk. Associations with *P* < .05 in the IVW analysis were considered statistically significant. Furthermore, genes showing discordant effect directions across the 5 MR statistical methods were excluded to ensure robustness. To validate the reliability of the MR results, comprehensive sensitivity analyses were conducted, including tests for heterogeneity (Cochran *Q* test), horizontal pleiotropy (MR-Egger intercept test), leave-one-out analysis, and Mendelian Randomization Pleiotropy Residual Sum and Outlier test. Results with evidence of horizontal pleiotropy (*P* < .05 in the MR-Egger intercept test) were excluded. All analyses were performed using R version 4.3.2, with the primary packages including TwoSampleMR, VariantAnnotation, and gwasglue. Visualization of results was carried out using ggplot2 and other plotting utilities.

### 2.8. Molecular docking

The molecular docking study was conducted to evaluate the binding interactions between the active compounds and target proteins. The three-dimensional structures of the target proteins, including von Hippel-Lindau (PDB ID: 1LM8) and inosine-5′-monophosphate dehydrogenase 2 (IMPDH2) (PDB ID: 6I0M), were retrieved from the RCSB Protein Data Bank. All water molecules and native ligands were removed from the protein structures using PyMOL version 2.3.0 (Schrödinger, LLC, New York ). The two-dimensional structures of the ligands (Jaranol [MOL000239], baicalein [MOL002714], β-ecdysterone [MOL012542], dehydrodiconiferyl alcohol 4,γ′-di-O-β-D-glucopyranoside_qt [MOL009029], and pontevedrine [MOL004224]) were obtained in SDF format from PubChem and subsequently converted into three-dimensional conformations using Chem3D (2020 release). These structures were then energy-minimized through molecular mechanics optimization. The prepared proteins and ligands were converted into the.pdbqt format using AutoDock Tools version 1.5.6. Docking simulations were performed using AutoDock Vina v.1.2.0 with the Lamarckian Genetic Algorithm under a semiflexible docking protocol. The exhaustiveness parameter was set to 8, and the maximum number of output conformations was set to 9. The grid box for von Hippel-Lindau was centered at coordinates (51.0, 20.0, and 127.2) with dimensions of 40 × 40 × 40 Å. For IMPDH2, the grid box was centered at (184.1, 293.4, and 377.0) with dimensions of 30 × 30 × 30 Å. Binding affinity was evaluated based on docking scores, with a total score ≥ 5.0 kcal/mol indicating strong binding. The resulting docking poses were visualized and analyzed using Discovery Studio 2021 Client (Dassault Systèmes, Vélizy-Villacoublay, France).

## 3. Results

### 3.1. Screening of active ingredients of GTD

Based on Traditional Chinese Medicine Systems Pharmacology Database and Analysis Platform (TCMSP) screening criteria (oral bioavailability ≥ 30%, drug-likeness ≥ 0.18) and supplementary literature review, 160 potential active ingredients of GTD were identified for OA treatment, including 14 from Astragalus, 57 from Salvia Miltiorrhiza, 22 from Eucommia, 0 from Angelica, 7 from Cuscuta, 47 from Corydalis Yanhusuo, 10 from Achyranthes, 0 from Cooked Rehmannia, and 3 from Poria Cocos.

### 3.2. Screening of component targets, disease targets, and intersection targets

**Figure 1.**
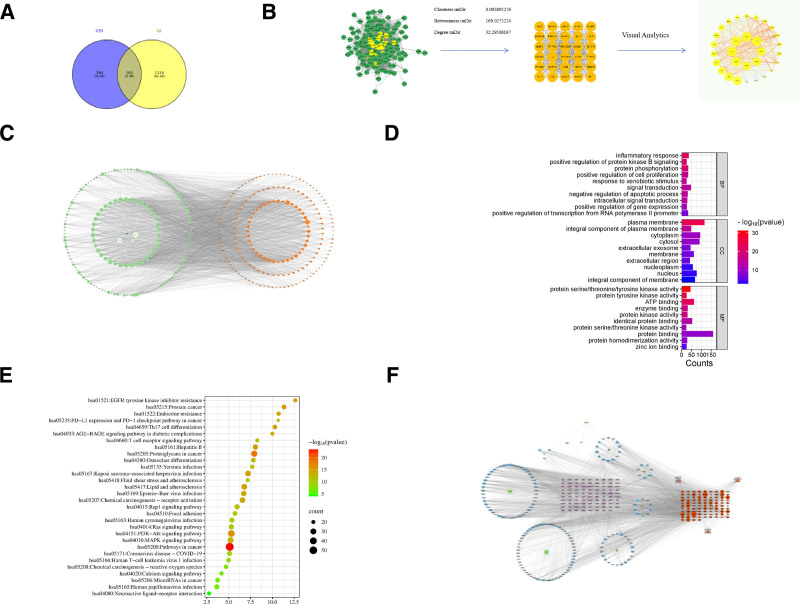
Results of network pharmacology analysis. (A) Venn diagram of component targets and disease targets of GTD. (B) PPI network of intersection targets of active ingredient and disease. (C) Active ingredient-target-disease target network. (D) GO function enrichment analysis. (E) KEGG pathway enrichment analysis. (F) Active ingredient-target-pathway network in GTD. GO = gene ontology, GTD = Gujian Tiaosui Decoction, KEGG = Kyoto encyclopedia of genes and genomes, PPI = protein–protein interaction.

A total of 575 active ingredient targets were identified by integrating targets from the TCMSP and SwissTargetPrediction databases. Additionally, 1500 disease-related targets were collected and deduplicated from the GeneCards and OMIM databases. These component and disease targets were then input into Venny 2.1 to identify overlapping targets (Fig. 1A), resulting in 185 intersection targets predicted to be relevant for drug action on the disease.

### 3.3. The construction of PPI network

The 185 intersection targets were imported into the STRING database with an interaction score set to the highest confidence level (0.900). Targets not involved in protein interactions were excluded to construct a PPI network. The resulting data were imported into Cytoscape 3.9.1 for further analysis and visualization (Fig. 1B). Nodes represent active ingredients and targets, while edges represent their interactions. Core targets (degree > 50) identified through PPI analysis included AKT serine/threonine kinase 1, hypoxia-inducible factor-1α (HIF1A), estrogen receptor 1, SRC proto-oncogene, non-receptor tyrosine kinase, epidermal growth factor receptor, mitogen-activated protein kinase 3 (MAPK3), matrix metalloproteinase 9 (MMP9), and signal transducer and activator of transcription 3 (STAT3).

### 3.4. Construction of component–target–disease networks

The intersection targets of active ingredients and disease targets were imported into Cytoscape 3.9.1 to construct a component–target–disease network (Fig. 1C). Nodes represent active ingredients, targets, and diseases, and edges represent their relationships. Topological analysis based on degree values reflected node importance, with higher values indicating greater significance. Key active ingredients among the 160 screened in GTD included β-ecdysterone, baicalein, dehydrodiconiferyl alcohol 4,γ′-di-O-β-D-glucopyranoside_qt, pontevedrine, and jaranol (Table 1).

**Table 1:** Important active ingredients of the combination.

TCM	Molecule name	Encoding	Degree
Radix Achyranthis Bidentatae	β-Ecdysterone	NX1	50.0
Radix Achyranthis Bidentatae	Baicalein	NX5	45.0
Cortex Eucommiae	Dehydrodiconiferyl alcohol 4, gamma′-di-O-beta-D-glucopyanoside_qt	DZ12	44.0
Corydalis Rhizoma	Pontevedrine	YHS1	44.0
Astragalus	Jaranol	HQ12	43.0

TCM = Traditional Chinese Medicine.

### 3.5. GO function and KEGG pathway enrichment analysis

Intersection targets related to the therapeutic effects of GTD on OA were imported into the Metascape database for GO functional enrichment analysis (*P* < .05), covering BP, cellular components, and molecular functions. As shown in Figure 1E, the primary BPs included inflammatory response, positive regulation of protein kinase B signaling, protein phosphorylation, regulation of cell proliferation, response to external stimuli, signal transduction, negative regulation of apoptosis, intracellular signaling, positive regulation of gene expression, and RNA polymerase II promoter transcription. Key molecular functions included integral component of membrane, protein serine/threonine/tyrosine kinase activity, protein tyrosine kinase activity, ATP binding, enzyme binding, protein kinase activity, protein homodimerization, and zinc ion binding. Major cellular components included plasma membrane, cytoplasm, extracellular exosome, extracellular region, and nucleoplasm. KEGG pathway enrichment analysis was further performed, with pathways ranked based on *P*-value and the number of enriched genes (Fig. 1F). Significantly enriched pathways included proteoglycans in cancer, MAPK signaling pathway, lipid and atherosclerosis, Kaposi sarcoma-associated herpesvirus infection, chemical carcinogenesis-receptor activation, among others. Bubble color indicates enrichment significance (red indicating higher enrichment), and bubble size represents the number of enriched genes.

### 3.6. Construction of active ingredient–target–pathway network diagram

Based on KEGG enrichment results, a chemical component–target–pathway network for GTD was constructed (Fig. 1D). Node size reflects degree value and correlation strength between components, targets, and pathways. To investigate the mechanism of GTD in treating OA, the top 3 enriched KEGG pathways (hsa04151: PI3K-AKT signaling pathway, hsa01100: metabolic pathways, and hsa05200: pathways in cancer) were analyzed at the BP level. An active ingredient–target–pathway network was subsequently constructed, providing evidence that GTD may serve as an effective treatment for OA.

### 3.7. HIF1A and IMPDH2 were viewed as risk factors for OA

**Figure 2.**
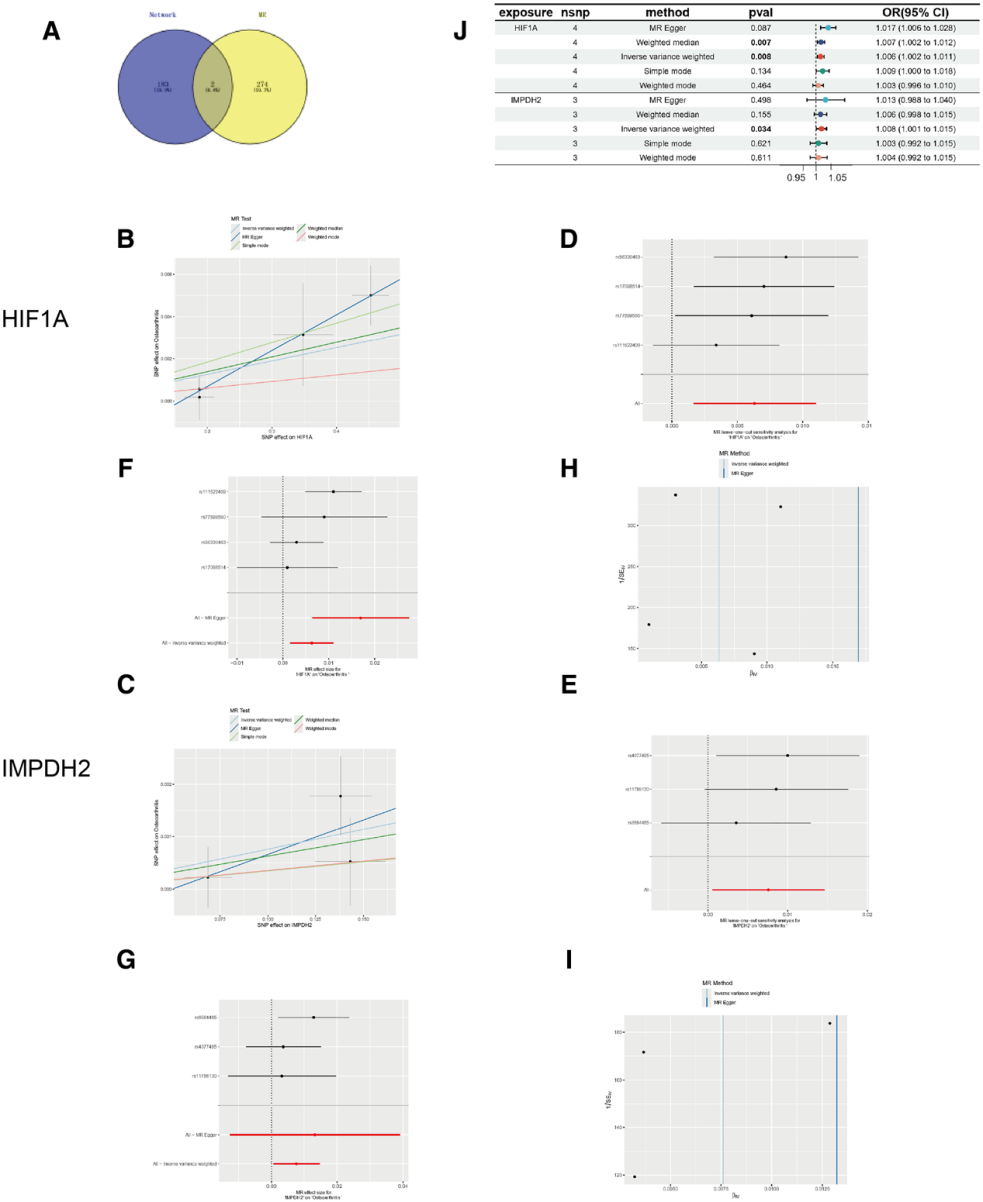
Results of Mendelian randomization analysis. (A) Venn diagram of network targets and MR targets. Mendelian randomization (MR) analysis and expression analysis for selecting 2 key targets in OA. (B and C) The scatter plot of the Mendelian randomization (MR) analysis for relationship of 2 key targets (B: HIF1A; C: IMPDH2) and OA. (D and E) Forest plots of the MR analysis for diagnostic of 2 key targets (D: HIF1A; W: IMPDH2) on OA. (F and G) Funnel plots of the MR analysis for 2 key targets (F: HIF1A; G: IMPDH2) on OA. (H and I) Leave-one-out analysis of the MR analysis for sensitivity analyses of 2 key targets (H: HIF1A; I: IMPDH2) on OA. (J) Forest diagram of MR analysis for 2 key targets of OA (F: HIF1A; G: IMPDH2). HIF1A = hypoxia-inducible factor-1α, IMPDH2 = inosine-5′-monophosphate dehydrogenase 2, OA = osteoarthritis.

A total of 276 target genes were identified through two-sample MR analysis exploring causal relationships between genes and OA. Among the 185 intersection targets of GTD active ingredients and OA, 2 key genes (HIF1A and IMPDH2) were identified using Venny 2.1 (Fig. 2A). These were significantly associated with OA (*P* < .05 using the IVW method; *P* > .05 in horizontal pleiotropy analysis; Table 2). Scatter plots revealed a positive correlation between HIF1A/IMPDH2 and OA (slope > 0; Fig. 2B and C). Forest plots further indicated that both genes are risk factors for OA (Fig. 2D and E). Funnel plots showed symmetric distributions, suggesting no directional pleiotropy (Fig. 2F and G). Leave-one-out analysis confirmed no significant deviation in IV effect estimates (Fig. 2H and I). Meta-analysis confirmed a positive causal relationship between both HIF1A (OR = 1.006, 95% CI: 1.002–1.011; *P* < .010) and IMPDH2 (OR = 1.008, 95% CI: 1.001–1.015; *P* < .010) with OA (Fig. 2J).

**Table 2:** Information for 2 targets causally associated with OA.

id.exposure	id.outcome	Method	nsnp	pval	pleio_pval
eqtl-a-ENSG00000100644	ebi-a-GCST90038686	IVW	4	0.007954171	0.167791685
eqtl-a-ENSG00000178035			3	0.034293075	0.730168987

IVW = inverse variance weighting, OA = osteoarthritis.

### 3.8. Analysis of molecular docking results

**Figure 3.**
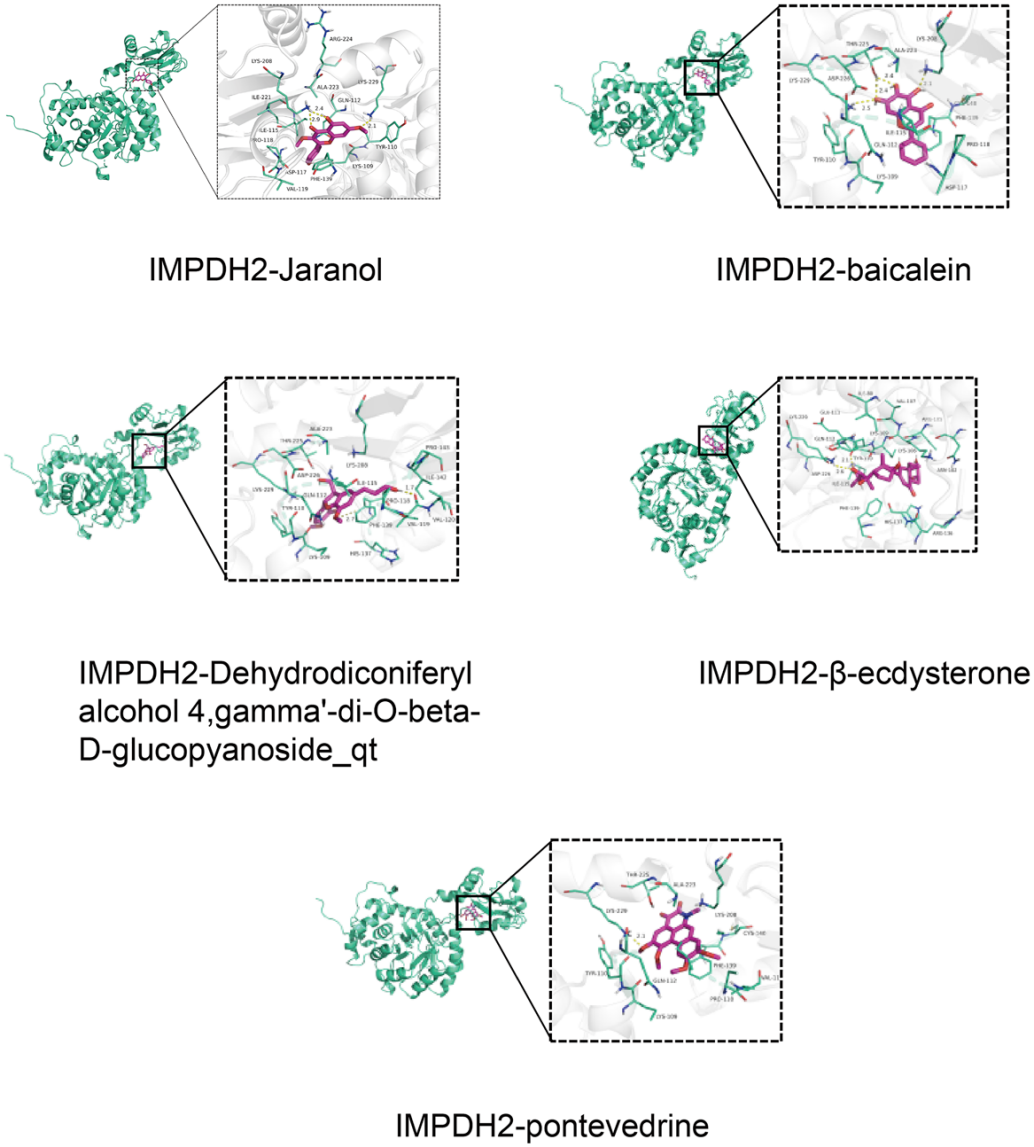
Docking model of key components and target HIF1A. HIF1A = hypoxia-inducible factor-1α.

**Figure 4.**
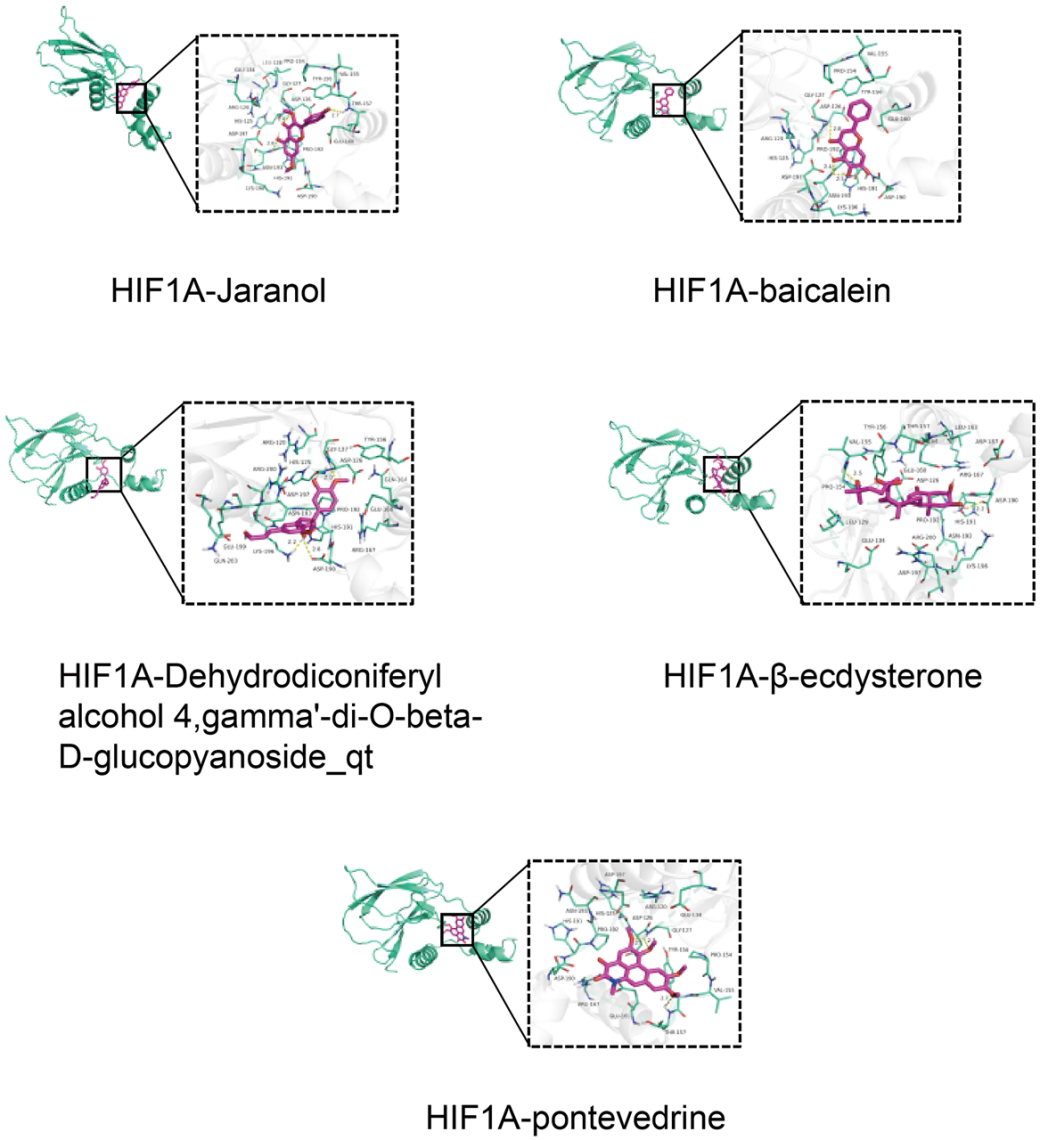
Docking model of key components and target IMPDH2. IMPDH2 = inosine-5′-monophosphate dehydrogenase 2.

All docking results demonstrated binding energies below −5 kcal/mol, indicating strong binding affinity between the compounds and target proteins (Table 3). Notably, baicalein (MOL002714) exhibited the highest binding affinity for IMPDH2 at −8.4 kcal/mol, while β-ecdysterone (MOL012542) showed the strongest binding to HIF1A at −7.5 kcal/mol. The complexes with the lowest binding energy for each ligand–target pair were visualized using PyMOL software. Further analysis of binding interactions revealed that these compounds form stable contacts with key functional residues within the active sites of HIF1A and IMPDH2. For example, baicalein formed hydrogen bonds with Thr196 and Glu244 in HIF1A (residues that contribute to the HIF-α degradation motif and are critical for its transcriptional activity). Similarly, β-ecdysterone engaged in hydrophobic interactions with Val228 and Ile256 in IMPDH2, a region implicated in substrate binding and catalytic function. Other compounds, including Jaranol and the dehydrodiconiferyl alcohol derivative, also exhibited favorable binding modes involving both polar and nonpolar interactions with functional residues of both targets (Figs. 3 and 4).

**Table 3:** Molecular docking results (kcal/mol).

Target point	PDB ID*	Active ingredient	Binding energy
HIF1A†	1LM8	β-Ecdysterone	−7.5
		Baicalein	−7.0
		Dehydrodiconiferyl alcohol 4, gamma′-di-O-beta-D-glucopyanoside_qt	−7.1
		Pontevedrine	−6.9
		Jaranol	−6.7
IMPDH2‡	6I0M	β-Ecdysterone	−7.4
		Baicalein	−8.4
		Dehydrodiconiferyl alcohol 4, gamma′-di-O-beta-D-glucopyanoside_qt	−7.9
		Pontevedrine	−7.9
		JARANOL	−7.9

* Protein Data Bank.

† Hypoxia-inducible factor-1α.

‡ Inosine-5′-monophosphate dehydrogenase 2.

## 4. Discussion

This study represents the first integrated application of network pharmacology and drug-target MR to systematically investigate the potential therapeutic mechanisms of GTD in OA. Through network pharmacology screening, we identified 185 overlapping genes between GTD’s active components and OA pathogenesis, suggesting their potential role as therapeutic targets. Enrichment analysis revealed significant enrichment in PI3K-AKT and MAPK signaling pathways. The PI3K-AKT pathway modulates OA progression through regulation of cell survival, metabolism, and autophagy. AKT phosphorylation (Ser473/Thr308) following pathway activation regulates downstream molecules including mTOR and FOXO, influencing chondrocyte apoptosis and matrix synthesis.^[15]^ The MAPK family (p38, JNK, and ERK) responds to inflammatory cytokines (IL-1β/TNF-α) or mechanical stress, regulating transcription factors (e.g., NF-κB, c-Jun) through phosphorylation cascades that mediate inflammation and matrix degradation.^[16]^ Crucially, MR analysis demonstrated that elevated expression levels of the HIF1A and IMPDH2 genes are positively causally associated with an increased risk of OA. This suggests that GTD may attenuate OA progression by suppressing the expression of these genes.

TCM offers unique advantages in conservative OA management through multi-target interventions and holistic regulation.^[17,18]^ Our findings further elucidate GTD’s molecular basis for OA treatment. The top 5 GTD components by degree value are: β-ecdysterone, baicalein, dehydrodiconiferyl alcohol 4, γ′-di-O-β-D-glucopyanoside_qt, pontevedrine, and jaranol. Existing literature supports the roles of β-ecdysterone and baicalein in OA: β-ecdysterone activates chondrocyte autophagy via PI3K/AKT/mTOR signaling,^[19]^ while baicalein inhibits synovial hyperplasia by suppressing pre-osteoblast proliferation, promoting apoptosis, reducing osteoblast differentiation, and impairing endothelial angiogenic capacity,^[20–22]^ with intra-articular administration demonstrating improved subchondral bone remodeling.^[23]^ Furthermore, our analysis implicates key therapeutic targets, including MMP9 and STAT3. MMP9 primarily degrades extracellular matrix components such as collagen and gelatin. Within OA pathogenesis, MMP9 overexpression is critically implicated in cartilage matrix degradation (a hallmark of disease progression). By degrading collagen and proteoglycans within the cartilage matrix, MMP9 disrupts cartilage structure and function, thereby accelerating OA development.^[24,25]^ This aligns with established evidence highlighting MMP9’s central role in OA pathophysiology. STAT3 is a pivotal transcription factor regulating diverse BP including cell proliferation, differentiation, apoptosis, and inflammatory responses. STAT3 critically contributes to OA pathogenesis by activating the NF-κB signaling pathway, which subsequently promotes inflammation and cartilage degradation, thereby driving disease progression.^[26]^ This corroborates prior reports of STAT3’s significant involvement in OA. Integrated pathway enrichment analysis indicates that GTD likely exerts its therapeutic effects against OA primarily through the modulation of key BP. These include inflammatory responses, positive regulation of protein kinase B (AKT) signaling, protein phosphorylation, membrane-associated functions, serine/threonine/tyrosine kinase activity, and protein tyrosine kinase activity.

Notably, our MR analysis provides the first causal genetic evidence identifying HIF1A and IMPDH2 as potential OA risk genes. HIF1A encodes HIF-1α, the master transcriptional regulator of cellular adaptation to hypoxia, which critically modulates chondrocyte autophagy, apoptosis, proliferation, and survival.^[27]^ HIF1A deficiency accelerates cartilage degeneration, manifesting as disrupted collagen architecture and significantly elevated MMP-13 expression.^[28]^ Our MR findings substantiate the causal relationship between HIF1A expression and OA pathology. IMPDH2, the predominant isoform in activated lymphocytes, mediates purine nucleotide synthesis.^[29]^ Significantly, immunosuppressants like mycophenolate mofetil treat inflammatory joint diseases (e.g., rheumatoid arthritis) precisely by inhibiting IMPDH2 activity.^[17,30]^ We thus propose that reducing IMPDH2 expression or activity may alleviate OA progression by suppressing intra-articular inflammation.

Molecular docking provided structural validation for network pharmacology and MR predictions. GTD’s key components (β-ecdysterone, baicalein, dehydrodiconiferyl alcohol 4, γ′-di-O-β-D-glucopyanoside_qt, pontevedrine, and jaranol) exhibited binding energies < −5 kcal/mol with both HIF1A and IMPDH2 targets, indicating favorable binding affinity and stable ligand–receptor complex formation. This further supports GTD’s capacity for effective and stable interaction with key therapeutic targets.

This study pioneers the integration of network pharmacology, drug–target MR, and molecular docking. Network pharmacology systematically predicted GTD’s mechanisms through a “multi-component, multi-target, multi-pathway” lens. MR analysis, leveraging genetic IVs to overcome confounding, to our knowledge, provided the first causal genetic evidence linking HIF1A and IMPDH2 to OA. Molecular docking structurally validated compound-target binding. This multidisciplinary strategy significantly enhances the depth and reliability of TCM formula mechanism research. Furthermore, we successfully identified HIF1A and IMPDH2 as potential key targets for GTD intervention in OA, with IMPDH2 representing a novel and understudied target in OA pathogenesis, offering fresh perspectives for understanding OA pathology and developing novel therapies. Finally, our workflow (spanning active component screening, target prediction, pathway enrichment, causal inference, and binding validation) establishes a comprehensive framework for elucidating the modern scientific basis of traditional empirical formulas like GTD.

Several limitations warrant consideration. First, network pharmacology predictions (components, targets, and pathways) rely on databases and algorithms, necessitating rigorous experimental validation. Predictive accuracy may be influenced by database completeness and algorithmic parameters. Second, MR results depend critically on the validity of instrumental IVs meeting relevance, independence, and exclusion restriction assumptions. Despite careful IV selection, potential biases from weak instruments or horizontal pleiotropy persist. Our MR focused on gene expression levels as the exposure, omitting posttranscriptional regulation, and protein activity alterations. Sample size and population heterogeneity in the OA GWAS data may also affect statistical power and generalizability. Most crucially, our core conclusions (particularly GTD’s therapeutic effect via HIF1A/IMPDH2 suppression and the predicted binding modes) remain computationally derived. Absence of validation through cell-based assays, animal models, or clinical trials constitutes the primary limitation of this study.

## 5. Conclusion

This integrated study employing network pharmacology, drug–target MR, and molecular docking systematically deciphers the molecular mechanisms underlying GTD intervention in OA. Our findings predict that GTD’s core bioactive components (e.g., β-ecdysterone, baicalein) likely exert therapeutic effects by targeting key molecules including HIF1A, IMPDH2, and AKT serine/threonine kinase 1, thereby modulating critical processes such as inflammatory responses and PI3K-AKT signaling pathways. Notably, this work provides pioneering genetically validated causal evidence (via MR analysis) supporting HIF1A and IMPDH2 as therapeutically relevant targets for OA. These results furnish a modern scientific foundation for understanding TCM interventions guided by the “Marrow Disease Theory” in OA management, while simultaneously illuminating novel therapeutic avenues for OA through the development of targeted agents against HIF1A or IMPDH2. Future research must prioritize rigorous validation of these predictions through in vitro and in vivo experimentation (e.g., assessing GTD or its bioactive constituents on HIF1A/IMPDH2 expression and downstream pathways in cellular and animal OA models) and well-designed clinical trials. Comprehensive investigation into GTD’s optimal formulation, effective dosing regimens, long-term efficacy, and safety profile remains essential.

## Acknowledgments

The authors sincerely thank the FinnGen consortium for providing high-quality GWAS and, which made this study possible. The authors also extend their gratitude to all participants and researchers who contributed valuable data to this study.

## Author contributions

**Conceptualization:** Yiwen Zhu.

**Data curation:** Xiaoming Wang.

**Funding acquisition:** Shirong Yang.

**Investigation:** Xiao Xiao, Jiao Situ.

**Methodology:** Qinguang Xu.

**Resources:** Jieji Zhang, Wenjie Xu.

**Software:** Denghui You, Yong Ju.

**Validation:** Xiaoming Wang, Yi Zhou.

**Visualization:** Jining Jiang.

**Writing – original draft:** Shirong Yang, Yiwen Zhu.

**Writing – review & editing:** Shirong Yang, Peijian Tong.
